# Radiology Imaging Adds Time and Diagnostic Uncertainty when Point of Care Ultrasound Demonstrates Cholecystitis

**DOI:** 10.24908/pocus.v9i1.16596

**Published:** 2024-04-22

**Authors:** David Cannata, Callista Love, Pascale Carrel, Trent She, Seth Lotterman, Felix Pacheco, Meghan Kelly Herbst

**Affiliations:** 1 University of Connecticut School of Medicine Farmington, CT USA; 2 Department of Emergency Medicine, Hartford Hospital Hartford, CT USA; 3 Department of Emergency Medicine, University of Connecticut School of Medicine Farmington, CT USA

**Keywords:** length-of-stay, Diagnostic imaging, point of care ultrasound, acute cholecystitis

## Abstract

Background: Point of care ultrasound (POCUS) is specific for acute cholecystitis (AC), but surgeons request radiology imaging (RI) prior to admitting patients with POCUS-diagnosed AC. Objectives: We sought to determine the test characteristics of POCUS for AC when performed and billed by credentialed emergency physicians (EPs), the accuracy rate of RI when performed after POCUS, and the time added when RI is requested after POCUS demonstrates AC. Methods: We performed a dual-site retrospective cohort study of admitted adult ED patients who had received biliary POCUS from November 1, 2020 to April 30, 2022. Patients with previously diagnosed AC, liver failure, ascites, hepatobiliary cancer, or cholecystectomy were excluded. Descriptive statistics and 95% confidence intervals for point estimates were calculated. Medians were compared using a Wilcoxon signed-rank test. Test characteristics of POCUS for AC were calculated using inpatient intervention for AC as the reference standard. Results: Of 473 screened patients, 143 were included for analysis: 80 (56%) had AC according to our reference standard. POCUS was positive for AC in 46 patients: 44 true positives and two false positives, yielding a positive likelihood ratio of 17.3 (95%CI 4.4-69.0) for AC. The accuracy rate of RI after positive POCUS for AC was 39.0%. Median time from ED arrival to POCUS and ED arrival to RI were 115 (IQR 64, 207) and 313.5 (IQR 224, 541) minutes, respectively; p < 0.01. Conclusion: RI after positive POCUS performed by credentialed EPs takes additional time and may increase diagnostic uncertainty.

## Background

Acute cholecystitis (AC) accounts for up to 25% of hospital surgery admissions and is associated with a mortality rate of 0.8% 1, 2. While consensus guidelines allow for diagnosis of suspected AC based on physical exam and laboratory findings, definitive diagnosis requires imaging findings consistent with AC 3. Radiology imaging (RI) modalities frequently used in the evaluation of AC include ultrasound, computed tomography (CT) and hepatobiliary iminodiacetic acid (HIDA) scan. Ultrasound and CT have comparable sensitivity and specificity, whereas HIDA has been shown to outperform these modalities but is more expensive and time-consuming 4, 5, 6. Given the favorable test characteristics of multiple potential imaging modalities that can be used to diagnose AC, a positive finding in one imaging test need not necessarily be confirmed by another imaging test. 

Ultrasound is considered the most appropriate initial imaging modality for a patient with suspected biliary pathology, per radiology society guidelines 7. Point of care ultrasound (POCUS) has demonstrated similar sensitivity and specificity for AC when compared with ultrasound performed by the Radiology Department (RADUS) 8, 9. Despite this, most surgeons still request additional RI modalities, such as RADUS and CT, to confirm AC before admitting the patient to their service. These requests may be related to surgeon attitudes and perspectives regarding the accuracy of POCUS, personal unfamiliarity with POCUS, and long-established practice patterns 10, 11, 12. 

POCUS diagnosis of AC by emergency physicians (EPs) has been shown to decrease ED length-of-stay 13. The 

addition of any RI modality after POCUS in the evaluation of AC results in an increased length of stay 14. This added time is clinically significant; delayed cholecystectomy has been associated with increased complications, mortality and costs 15. Disagreement between POCUS and subsequent imaging may paradoxically increase diagnostic uncertainty for AC. 

This study has three aims: first, to determine the test characteristics of POCUS for AC when performed and billed by credentialed attendings in two ED settings; second, to determine the accuracy rate of RI following a positive POCUS using inpatient intervention for AC as the reference standard; and third, to determine the time added when subsequent RI is requested after POCUS demonstrates AC. We hypothesized that POCUS performed by credentialed EPs is accurate when positive for AC, and that the accuracy of POCUS is greater than that of subsequent RI for evaluation of AC in this setting. Describing the value or lack thereof of subsequent imaging after a positive POCUS and quantifying the associated potential time delay may lead to improved clinical practice and more efficient hospital admissions processes. 

## Methods

This was a dual-site retrospective cohort study at two hospitals of all patients presenting to the ED who received a biliary POCUS examination by a credentialed EP during their ED visit. The study data was collected over an 18-month period, from November 1, 2020 to April 30, 2022. Institutional Review Board (IRB) approval was obtained from the study institutions. The study was deemed exempt and consent was waived. 

### Study Setting and Population 

Study data was obtained from two sites. The first is a community hospital designated Level III Trauma Center in a suburban area, with 38,000 annual ED visits and a ten percent admission rate. It is the primary site of a four-year university-based medical school and a community site for an emergency medicine residency program. The hospital has four credentialed biliary ultrasound attendings and two Sonosite X-porte (FUJIFILM Sonosite, Inc, Bothell, WA) ultrasound machines. The second is an urban Level I Trauma Center with approximately 120,000 annual ED visits and a 30 percent admission rate. It is the primary clinical site for an emergency medicine residency and has six credentialed biliary ultrasound attendings and six ultrasound machines: three Sonosite X-Porte, Sonosite M-Turbo (FUJIFILM Sonosite, Inc, Bothell, WA), Mindray M-9 (Mindray, Shenzhen, China) and Philips Sparq (Philips Healthcare, Andover, MA). 

### Patient Selection 

All admitted patients 18-years-old who had a biliary POCUS examination performed and billed at either hospital during the study period were included. Biliary POCUS examinations may be conducted in both sites for diagnostic or educational purposes. For a scan to be billed, it must be a diagnostic scan performed by a credentialed EP with saved images and an accompanying interpretation. It is the policy at our institutions that when working with residents, credentialed EPs supervise the acquisition and interpretation of POCUS scans acquired by residents. Attendings then attest to these when they document and sign the POCUS interpretation. Credentialed EPs are attending-level physicians designated by the ED ultrasound director according to American College of Emergency Physicians (ACEP) Guidelines 16. Patients with liver failure, ascites, or hepatobiliary cancer were excluded due to the effect these diagnoses can have on the gallbladder that may complicate diagnosis. Patients with prior partial or complete cholecystectomy, patients sent to the ED with known cholecystitis, and patients who left against medical advice before their active biliary workup was complete, were also excluded. If patients meeting inclusion criteria had repeat ED visits during the study period, only the most recent visit was included. 

### Data Collection 

Using an automated electronic medical record (EMR) report, a list of all patients who received a billed biliary POCUS examination in the ED during the study period was used as the basis for the chart review. Patients discharged from the ED were removed, exclusion criteria were applied, and a structured chart abstraction was performed by four independent investigators following a one-hour educational session on a specific systematic approach to collecting data variables from the EMR. There was a prespecified random 10% overlap of charts reviewed to ensure consistency and accuracy in data abstraction amongst the four investigators. 

The time stamp of the first radiology report was used as the RI interpretation time. The acquisition time of the last POCUS image acquired was used as the POCUS interpretation time, since the physician performing POCUS interprets obtained images simultaneously 17. 

POCUS was positive for AC if there was both an obstructing stone in the gallbladder neck and a positive sonographic Murphy’s sign (SM), as these findings are the minimum criteria for AC at the two study sites and have been shown to be sensitive and specific in ultrasound-guided diagnosis of AC 18, 19. Sludge was considered equivalent to stone(s), and the cystic duct was considered contiguous with the neck given both appear similarly on ultrasound. Investigators abstracted the presence of gallstones, SM, wall thickening, and pericholecystic fluid according to POCUS procedure documentation from the EMR. The standard POCUS interpretation documentation did not require the physician to indicate the location of gallstones when present. For this reason, two credentialed EPs with ultrasound fellowship training blinded to chart variables and patient outcomes independently reviewed the POCUS images of patients with documentation of both SM and stones present. Next, they came to a consensus on whether any of the stones present were in the neck to identify cases of sonographic AC in our study population. RI was positive for AC if the first radiology report included any mention of concern, suspicion for, or findings consistent with AC. Patients with cholelithiasis but no mention of cholecystitis were considered negative for AC on RI. We only considered the first RI modality performed if there was more than one. 

The reference standard for AC diagnosis was defined as active intervention targeted at AC during the hospital admission associated with the ED visit. Specifically, this was cholecystectomy demonstrating cholecystitis on the pathology report, percutaneous cholecystostomy tube placement, or inpatient intravenous antibiotic therapy targeted towards pathogens suspected to cause AC with delayed cholecystectomy demonstrating cholecystitis on the pathology report. Patients who did not receive any of these interventions during the admission that followed the initial ED presentation were considered to not have AC by our reference standard. 

### Measuring Outcomes and Data Analysis 

We used Microsoft Excel (Microsoft Corp) and MedCalc Statistical Software version 19.7.4 (MedCalc Software LTd, Ostend, Belgium; https://www.medcalc.org; 2022) for descriptive statistics and statistical calculations. Sensitivity, specificity, and positive and negative likelihood ratios of POCUS for cholecystitis (dichotomized to present or absent) were calculated, using inpatient intervention as defined above as the reference standard. An accuracy rate for RI following positive POCUS was calculated by dividing the true positive RI by the total number of RI examinations. Medians were compared using a Wilcoxon signed-rank test. 

## Results

**Figure 1:**
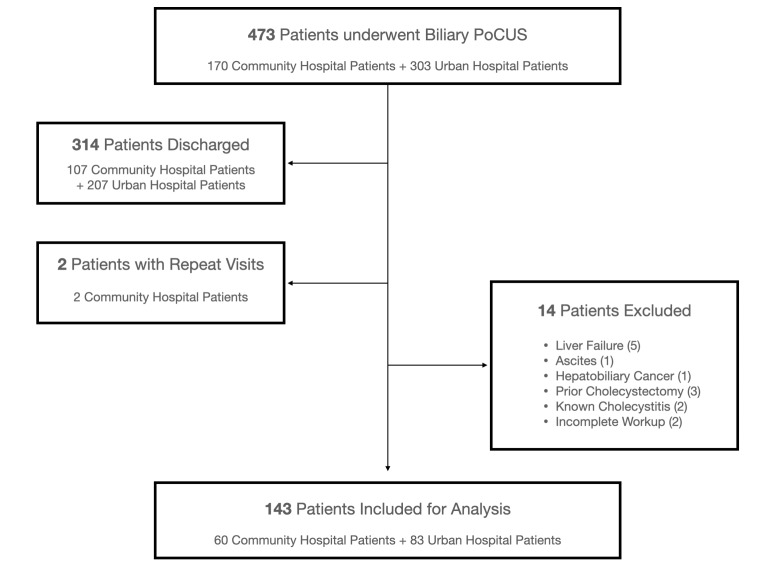
Patient flow chart. POCUS = point of care ultrasound

Overall, 473 patients across both sites underwent biliary POCUS billed by a credentialed attending in the ED, 170 patients from the community hospital and 303 patients from the urban trauma center. Two patients (both from the community hospital) had recurrent ED visits during the study period where only the most recent visit for each was included. Three-hundred-fourteen patients were discharged from their ED visits and 14 patients met exclusion criteria, leaving 143 patients for analysis (Figure 1). There was 100% agreement across all investigators for overlapping data. 

ummarizes patient demographics. Of the 143 patients, 80 (56%) had AC according to our reference standard: 69 had immediate cholecystectomy with subsequent pathology demonstrating cholecystitis, 7 had percutaneous cholecystostomy tube placement, and 4 received intravenous antibiotics with delayed cholecystectomy demonstrating cholecystitis. Of the 143 POCUS examinations, 62 had a positive SM, of which 57 had gallstones, and among those, 46 had a stone in the neck of the gallbladder. Among these 46 patients with positive POCUS for AC, two were falsely positive. POCUS had a specificity of 96.8% (95% CI 89.0-99.6) and positive likelihood ratio of 17.3 (95% CI 4.4-68.7) for AC. Table 2 summarizes POCUS test characteristics.

**Table 1:** Patient Demographics According to Hospital Site.

	Hospital X* (N=60)	**Hospital Y (N=83)**	**Total** **(N=143)**	**Significance** p-value
Age – yr.	57.3	51.9	54.2	0.100
Female Sex - %	60.0	78.3	70.6	0.025
**Race/Ethnicity – no. (%)**				**†**
Asian	4 (6.7)	3 (3.6)	7 (4.9)	0.453
Black	5 (8.3)	8 (9.6)	13 (9.1)	1.00
Hispanic	6 (10)	31 (37.3)	37 (25.9)	<0.001
White	44 (73.3)	36 (43.4)	80 (55.9)	<0.001
Other	1 (1.7)	5 (6.0)	6 (4.2)	0.401
Acute Cholecystitis – no. (%)	28 (46.7)	52 (62.7)	80 (55.9)	0.063

† Race and ethnicity were obtained from the medical record registration information.

* Hospital X refers to the community hospital; Hospital Y refers to the urban Level 1 trauma center.

**Table 2:** Test Characteristics for Point of Care Ultrasound in the Diagnosis of Cholecystitis

**Test Characteristic**	Value (N = 143)*	**95% Confidence Interval**
Sensitivity	55.0%	43.5-66.2%
Specificity	96.8%	89.0-99.6%
Positive Likelihood Ratio	17.3	4.4-68.7
Negative Likelihood Ratio	0.46	0.36-0.59
Positive Predictive Value	55.9%	47.4-64.2%
Negative Predictive Value	62.9%	57.0-68.4%
Accuracy	73.4%	65.4-80.5%
*Based on 44 true positives, 2 false positives, 36 false negatives, and 61 true negatives		

Additional radiologic imaging (RI) was performed after POCUS in the ED for 122 of the total 143 patients (46 at the community hospital and 76 at the urban trauma center). Forty-five patients had subsequent CT scans, 76 patients had subsequent RADUS, and one patient had an MRI. 

**Figure 2:**
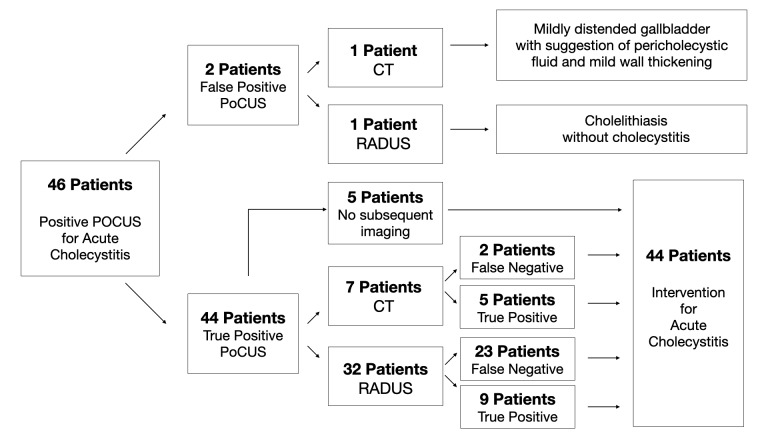
Figure 2. Visual summary of subsequent imaging and intervention among patients with positive biliary point of care ultrasound. POCUS, point of care ultrasound; RADUS, Radiology-performed ultrasound; CT, computed tomography.

Among the 46 positive POCUS examinations, 41 had subsequent RI (8 CT, 33 RADUS). Of these 41 patients, 25 were falsely negative for AC according to our reference standard, yielding an accuracy rate of 39.0% (95% CI 24.2-55.5). Figure 2 summarizes the sequence of events from positive POCUS to subsequent RI and AC intervention. 

Two patients had a false positive POCUS examination for AC according to our reference standard, with a subsequent true negative RADUS examination. The first patient presented with sepsis and transaminitis, and her blood cultures grew extended-spectrum beta-lactamase-producing Escherichia coli. A magnetic resonance cholangiopancreatography study showed gallstones and gallbladder distension without ductal dilatation or choledocholithiasis. Repeat blood cultures were negative, and she was discharged on antibiotics. Her bacteremia was thought to be due to transient choledocholithiasis. The second patient presented in septic shock with diffuse abdominal pain. POCUS demonstrated a gallstone in the neck of the gallbladder with wall edema. CT revealed diffuse pneumoperitoneum of unclear origin with diffuse mesenteric edema and intra-abdominal free fluid, as well as a distended gallbladder with pericholecystic fluid and wall thickening, but no pneumobilia to suggest biliary source for pneumoperitoneum. She underwent exploratory laparotomy, sigmoid colectomy, colostomy creation and umbilical herniorrhaphy. She was discharged with a diagnosis of pneumoperitoneum of unknown etiology. 

The median time from ED arrival to POCUS and ED arrival to subsequent RI for all patients who received both a POCUS and RI (n = 122) was 115 (IQR 64, 207) minutes and 314 (IQR 224, 541) minutes respectively (p < 0.01 for the difference). Among patients with AC on POCUS (n = 46), median time from ED arrival to POCUS and ED arrival to subsequent RI was 107 (IQR 53, 183) minutes and 313 (IQR 227, 471) minutes respectively (p < 0.01). The RI result was available after the POCUS result in this group of patients following a median interval of 175 (112, 358) minutes, or 2.9 hours. 

Regardless of the RI result (i.e., true positive or false negative), the median ED length of stay, hospital length of stay, and time to cholecystectomy were not significantly different among the patients who had a positive POCUS and subsequent RI (p = 0.34, p = 0.45, and p = 0.20, respectively). 

## Discussion

Our results demonstrate that RI not only adds hours to the ED workup, but also adds diagnostic uncertainty in the setting of a positive POCUS examination for AC. Prior studies of POCUS for AC have reported test characteristics of POCUS, time added by subsequent RI, and agreement between POCUS and subsequent RI. To our knowledge, none have focused on the utility of additional RI after POCUS demonstrates cholecystitis, using inpatient hospital intervention and pathology reports as the reference standard for AC. Our patients with a positive POCUS for AC overwhelmingly had AC-directed inpatient management even with negative subsequent RI. These results suggest that subsequent RI is of limited utility in the case of a positive POCUS for AC. 

Our results compare favorably with recent literature. For example, Zitek et al., Evans et al., and Hilsden et al. found a similarly high specificity of POCUS for AC using a less robust reference standard when performed by novice to experienced physicians; whereas our result was for POCUS performed and billed by credentialed providers using AC inpatient intervention 14, 20, 21. Since credentialed attendings, not learners, are the ones making clinical decisions using POCUS, their results are most relevant to patient care and clinical decision making. 

Hilsden et al. and Evans et al. reported time added by performing additional imaging. Our results confirmed this finding and suggested that this added time is even more substantial when POCUS is performed by credentialed physicians 14, 21. In our study, the time added by ordering additional RI after positive POCUS was 41% higher than that of these studies. 

Although there are multiple findings that can be seen on POCUS in the setting of AC, there is notable variability regarding the minimum criteria to define AC on POCUS. The presence of an objective finding (obstructing stone in the neck of the gallbladder) plus a local sign of inflammation (SM) as used in this study meet criteria for AC according to validated guidelines and also carry a high positive predictive value for AC 3, 18, 19. While comparable to recent studies where POCUS was considered positive for AC based on documented interpretation alone, without review of specific imaging findings 9, 20, 21, our specificity was greater than that reported by a study from over a decade ago with similar, but less stringent, criteria for AC (gallstones plus either gallbladder wall thickening, pericholecystic fluid, or SM) 8. The improvement of published test characteristics of POCUS over time can likely be attributed to increased POCUS utilization, more strict training milestones and quality standards for credentialed EPs, and advancements in ultrasound technology. 

We were surprised at the high false negative rate of RI for AC, particularly of RADUS, given that most clinicians use RADUS as a gold standard for diagnosis. While this finding was likely much higher in our sample of patients with positive POCUS for AC than it would be among the general population, it may explain why so many imaging studies are ordered to diagnose AC: imaging in isolation is not ideal for AC diagnosis 20. An inherent difference between POCUS and RI is the degree of separation between the physician and the patient. With POCUS, there is no separation of physician from the patient, allowing real time correlation of findings (including a positive SM when present) with patient presentation. However, there are many degrees of separation between the physician and patient with RI, making it more difficult to correlate imaging findings with the clinical picture. This can lead to diagnostic uncertainty, especially among cases of early cholecystitis, where there are not yet wall changes or pericholecystic fluid 22. This suggests that when POCUS is positive, particularly with the specific criteria used in this study, additional RI does not add clarity to a diagnosis and instead may add ambiguity and complicate disposition decisions. Despite additional RI often disagreeing with a positive POCUS, patients in our sample eventually had AC-directed inpatient management without any significant difference in ED length of stay, hospital length of stay, or time to cholecystectomy. This was true regardless of the findings of subsequent RI. We believe this could have been because the patients with a positive POCUS for AC had other evident signs that may have convinced admitting providers to treat for AC. It may have been that the surgeons making the decision to take these patients to the operating room relied on patient history, serial examinations, symptomatic response to oral intake, and laboratory results rather than additional RI alone, even if that RI was negative for AC. It is possible that additional imaging was requested and performed for operative planning more so than to diagnose AC 23. Because it was not an aim of our study to look at the rationale for definitive AC management, much of this is left to speculation. Regardless, the fact that subsequent RI did not affect the time nor the rate of AC management further questions its utility. For these reasons, we feel additional RI should be reserved for cases where initial imaging results are negative or equivocal and clinical suspicion remains high. 

From the perspective of an EP, the goal of care for every patient with AC is timely intervention and prompt disposition to an admitting service. Though there are many variables in ED length of stay, additional RI adds time and uncertainty to diagnosis. The use of POCUS in identifying AC quickly and accurately has the potential to expedite the admission process and prevent delays in intervention that may impact patient outcomes. 

### Limitations

There are several limitations in this study. Because it is a retrospective chart review, the findings are based only on what is documented in the chart. As such, it is difficult to ascertain with certainty the precise clinical picture, all the findings made by the EP, and the extent and nature of discussions between the EP and the consultants. We attempted to keep the POCUS definition objective by conducting an independent review for gallstone location to determine the presence of a stone in the neck, however, we were reliant on the chart for the presence of stones and SM. Our POCUS definition was not intended to detect acalculous cholecystitis, given its rare occurrence, tendency to affect patients with critical illness, and often requirement for additional imaging. 

The design of this study is based on ultrasound imaging, without accounting for the myriad of other factors that may impact a clinician’s decision to delay intervention or surgical admission. 

Although two sites were included, both are in the same state and region, so results may not be generalizable to other states, regions, or settings. Furthermore, there may have been variability in the time intervals studied according to site. If there were longer ED lengths of stay at the urban trauma center, for example, this may have obscured a difference in length of stay due to the relative contribution of each site, even though we utilized medians with IQRs to mitigate this. 

We did not compare ED length of stay when POCUS was performed alone versus when additional RI were performed, given POCUS was not generally considered diagnostic outside of the ED at our institutions. Too few patients had POCUS alone to adequately power this comparison. We did not include patients diagnosed with AC who did not receive POCUS and did not focus on patients who were diagnosed with AC with an initial negative POCUS. We directed our analysis on the utility of additional RI in the setting of a positive POCUS for AC, which is a clinical scenario where the burden of disposition should not hinge on further, unnecessary testing to diagnose AC. Future prospective studies are needed to look at time and costs saved when POCUS is performed alone, as well as the rate of potential surgical complications that might result from delays to admission among patients with AC. 

## Conclusions

POCUS performed and billed by ultrasound-credentialed attendings in the ED is specific and carries a high likelihood ratio for AC. RI after POCUS in this setting may detract from true positive results, takes additional time, and may not be required on a routine basis; rather, it should be reserved for complicated presentations or inconclusive POCUS studies. Further research is needed to determine which patients would benefit from RI after POCUS. 

## Disclosures

Acknowledgements: The authors would like to thank Jonah Haber MS for his contributions to an earlier version of this work; João Delgado MD for his assistance in reviewing and editing the manuscript; and James Grady PhD for his statistical assistance. 

## Funding 

None

## Other disclosures/Conflicts of interest

None 

## Ethical approval

This study was approved by the University of Connecticut Health Center Institutional Review Board (study number 22X-277-1, approved on May 4, 2022) and Hartford Healthcare Institutional Review Board (study number D-HHC-2022-0119, approved on May 18, 2022). 

## Disclaimers

None 
